# A Two-Dimensional Model of the Colonic Crypt Accounting for the Role of the Basement Membrane and Pericryptal Fibroblast Sheath

**DOI:** 10.1371/journal.pcbi.1002515

**Published:** 2012-05-24

**Authors:** Sara-Jane Dunn, Paul L. Appleton, Scott A. Nelson, Inke S. Näthke, David J. Gavaghan, James M. Osborne

**Affiliations:** 1 Department of Computer Science, University of Oxford, Oxford, United Kingdom; 2 Microsoft Research Ltd., Cambridge, United Kingdom; 3 College of Life Sciences, University of Dundee, Dundee, United Kingdom; 4 Oxford Centre for Integrative Systems Biology, Department of Biochemistry, Oxford, United Kingdom; University of Notre Dame, United States of America

## Abstract

The role of the basement membrane is vital in maintaining the integrity and structure of an epithelial layer, acting as both a mechanical support and forming the physical interface between epithelial cells and the surrounding connective tissue. The function of this membrane is explored here in the context of the epithelial monolayer that lines the colonic crypt, test-tube shaped invaginations that punctuate the lining of the intestine and coordinate a regular turnover of cells to replenish the epithelial layer every few days. To investigate the consequence of genetic mutations that perturb the system dynamics and can lead to colorectal cancer, it must be possible to track the emerging tissue level changes that arise in the crypt. To that end, a theoretical crypt model with a realistic, deformable geometry is required. A new discrete crypt model is presented, which focuses on the interaction between cell- and tissue-level behaviour, while incorporating key subcellular components. The model contains a novel description of the role of the surrounding tissue and musculature, based upon experimental observations of the tissue structure of the crypt, which are also reported. A two-dimensional (2D) cross-sectional geometry is considered, and the shape of the crypt is allowed to evolve and deform. Simulation results reveal how the shape of the crypt may contribute mechanically to the asymmetric division events typically associated with the stem cells at the base. The model predicts that epithelial cell migration may arise due to feedback between cell loss at the crypt collar and density-dependent cell division, an hypothesis which can be investigated in a wet lab. This work forms the basis for investigation of the deformation of the crypt structure that can occur due to proliferation of cells exhibiting mutant phenotypes, experiments that would not be possible *in vivo* or *in vitro*.

## Author Summary

At the onset of colorectal carcinogenesis, marked changes can be observed in the structure and dynamics of the crypts of Lieberkühn. These test tube shaped glands regularly punctuate the surface of the gut and are lined with a monolayer of epithelial cells which divide and migrate upwards to renew the intestinal surface every few days. The process by which the crypt structures breakdown, and the compliant environment that can be subsequently provided to mutated cells to allow the formation of adenomatous growths, is not yet well characterised. A limiting factor in the understanding of this process is the ability to observe easily the initial changes that occur, and which are necessary to disrupt the normal behaviour of the system. However, a predictive, theoretical model of the crypt that mimics the geometry and the tissue architecture can be used to perform *in silico* experiments and further such understanding. A model is introduced here that addresses the tissue structure of the crypt, and the stability it provides to the epithelial layer, while remaining deformable and without imposing a fixed geometry.

## Introduction

**Figure 1:**
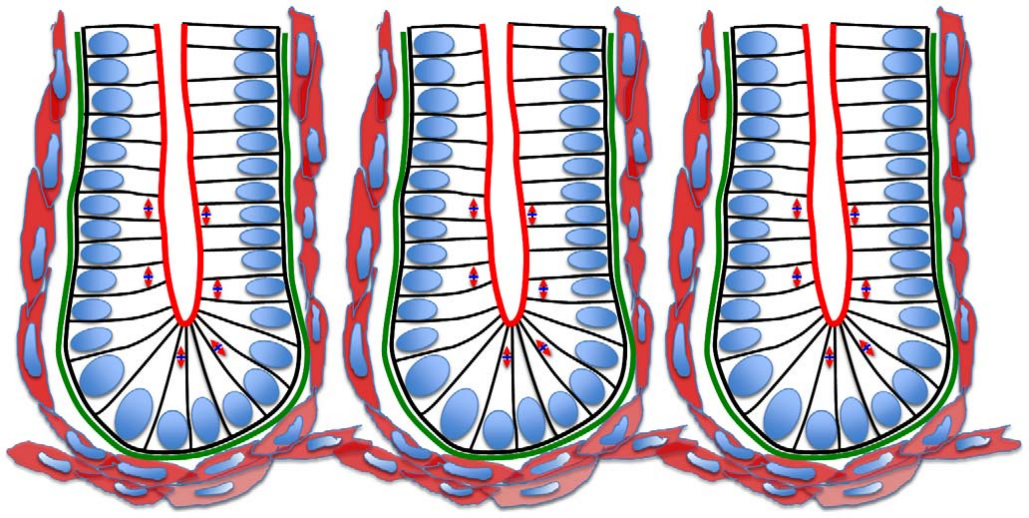
A schematic illustration of the crypts of Lieberkühn. Neighbouring crypts are closely packed, and each is composed of a monolayer of columnar epithelial cells (nuclei shown in blue). The apical surface of each cell faces the lumen (the brush border of the cells is indicated in red), and the basal surface is in contact with the basement membrane (green). The arrows in the cells indicate the changing mitotic spindle alignment moving up the crypt axis, illustrating the switch from mostly asymmetric to symmetric cell division. Lastly, the surrounding pericryptal fibroblasts are shown in pink, myofibroblasts that provide chemical and mechanical factors for normal crypt structure. The surrounding musculature forms a basket beneath the base of each crypt.

Colorectal cancer (CRC) is one of the leading causes of cancer-related death worldwide, demanding a response from scientists and clinicians to understand its aetiology and develop effective treatment. CRC is thought to originate via genetic alterations that cause disruption to the cellular dynamics of the crypts of Lieberkühn, test-tube shaped glands located in the small and large intestine, which are lined with a monolayer of epithelial cells (see Fig. 1). A delicate balance of cell division, migration and death is coordinated in the crypts to renew the epithelial layer every few days [1], [2]. The regular upward migration and removal of cells from the crypt provides a frontline defense mechanism against potential damage from mutated cells, which are prevented from remaining in the crypt long enough to do significant damage. However, if cells accumulate genetic mutations that alter migration velocity or provide resistance to apoptosis cues, then such cells acquire the ability to persist and multiply in the crypts. This alone can increase stress on the walls of the crypts, but the problem will be aggravated if such cells acquire additional mutations that increase proliferation, or alter cell-cell adhesion. In turn, the increased stress can cause the walls of the crypt to buckle. Dysplastic crypts allow the formation of a benign adenoma if mutated cells do not leave the crypt as they should, but rather persist and proliferate in a localised area. Over time and via accumulated mutations, these growths can progress to a malignant lesion that can break through to the underlying tissue stroma, and so aid metastasis.

The dynamic cell properties that are required to initiate crypt buckling are poorly understood, as it is difficult for biologists to observe experimentally, either *in vivo* or *in vitro*, the initial changes in this sequence of events. For example, the organoids grown in culture by Sato *et al.* [3], while recapitulating the crypt geometry, have not yet been compared in detail with crypts *in situ*. The organoids lack some of the forces that are present *in vivo*, and the cells themselves do not migrate. However, performing *in silico* experiments using a computational model of the crypt *in situ* could highlight the conditions required for buckling to occur, and so provide crucial insight into the tissue-level effects of genetic mutations that lead to CRC. To achieve reliable predictions of the breakdown of the crypt structure that occurs at the onset of carcinogenesis, such a theoretical model of the crypt must link processes occurring at the subcellular, cellular and tissue levels. The model must also take into account the tissue structure and geometry. While a fully comprehensive model is not yet realised, this work concerns a key step in the development of a predictive, computational model of the crypt which defines structural components in accordance with the tissue architecture that is observed experimentally, and reported here. These elements are incorporated into a crypt model which also addresses the coordination of cell division, polarity, differentiation and apoptosis.

As depicted in Fig. 1, individual crypts are closely packed, surrounded and separated by connective tissue. Each crypt is lined with an epithelial monolayer that consists of contiguous cells separated from the connective tissue and musculature by the basement membrane, the primary contact site for epithelial cells to the extracellular matrix. Below the basement membrane are myofibroblasts that provide chemical and mechanical factors for normal crypt structure. There is an established proliferative hierarchy of cells within the epithelial layer: stem cells reside at the base and divide to produce transit amplifying cells, which migrate up the crypt and perform several symmetric divisions before terminally differentiating. The polarised epithelial cells are oriented with the apical membrane facing the crypt lumen and, during symmetric division, mitotic spindles align parallel to the tissue layer [4], [5]. Consequently a cell places its daughter cell next to it within the plane and the monolayer is maintained throughout growth. Asymmetric division occurs as a consequence of the perpendicular alignment of the mitotic spindle.

Differentiated epithelial cells, upon having reached the crypt collar, undergo apoptosis and/or are shed into the lumen [6], [7]. This process permits the renewal of the epithelial layer every few days. In addition to this, a form of programmed cell death, anoikis, is triggered when there is inadequate adhesion of the epithelial cells to the extracellular matrix [8], with detachment inducing apoptosis [9]. Functioning correctly, this maintains tissue homeostasis by restricting proliferation to the monolayer, thereby averting dysplasia, and by preventing cells from reattaching in another location and resuming growth.

The Wingless/Int (Wnt) signalling pathway is involved in the control of cell proliferation, migration, differentiation and adhesion in the crypts [10], [11]. The Wnt signalling pathway is required to maintain the stem cell compartment in the crypt, and so is crucial to stem cell renewal and differentiation [12]. Moreover, it has been observed that there is a spatial gradient of extracellular Wnt signalling factors along the vertical crypt axis, which suggests a localised source of diffusible Wnt factors in the stroma that surrounds the crypt base, and leads to the hypothesis that a Wnt gradient may be responsible for the observed proliferative hierarchy [13]. As described in Van Leeuwen *et al.* [10], cells in the presence of high concentrations of Wnt cycle for longer than those exposed to low Wnt and hence cells at the base of the crypt are expected to remain proliferative.

A number of mathematical models exist that aim to describe specific aspects of crypt behaviour, from Wnt dependent ordinary differential equation (ODE) cell cycle models that govern mitosis of individual cells [14], [15], to cellular automata and lattice-free mechanical models of cell proliferation and migration [16]–[19]. These ideas have been combined in a multiscale model that has been used to investigate clonal expansion and the disruption of crypt homeostasis that forms the first step in colorectal carcinogenesis [15]. However, these models restrict the domain of investigation by prescribing a rigid, cylindrical geometry to the crypt, and are limited by simplifying the tissue structure without considering the basement membrane and surrounding stroma. This prevents such models from realistically examining the tissue-level effects of abnormal cell behaviour.

There also exist models that seek to describe crypt buckling. Edwards and Chapman (2007) [20] present a continuum representation of the crypt, modelled as a growing beam, while Drasdo and Loeffler (2001) [21] apply an off-lattice overlapping spheres model to describe a two-dimensional (2D) chain of deformable circles such as occurs during blastula formation, and then restrict this to a U-shape for modelling the crypt. These models commonly assign a bending stiffness to the layer, and predict that buckling will occur if growth by cell division is not adequately matched by this force. Edwards and Chapman generalise cell division events and so do not implement a specific cell cycle model to govern mitosis, though possible in this framework, and none of these examples take into account the deformation of the surrounding tissue stroma. More recently, Nelson *et al.* (2010) [22] extended the continuum model due to Edwards and Chapman [20] to investigate how growth of an epithelial monolayer constrained to a flexible substrate can recapitulate the geometry of the crypt, and Hannezo *et al.* (2011) [23] present a model of the intestinal crypt-villus architecture arising from a buckling instability in a proliferating epithelial monolayer lying on an elastic substrate.

A three-dimensional (3D) agent-based crypt model was proposed by Buske *et al.* (2011) [24], which defines lineage specification and differentiation according to threshold-dependent rules that correspond to the effects of Wnt- and Notch- signalling. This model addresses the pedigree concept of cell stemness, and reproduces the spatio-temporal organisation experimentally observed in the crypt without assuming an explicit stem cell population. For this purpose, the authors model the basement membrane as a fiber network with a defined local radius for each cell position, which thereby defines a fixed crypt geometry. Consequently, it is not possible to follow any deformation of the crypt structure, and the authors do not include more sophisticated subcellular pathways that determine cell division or fate.

Also relevant to the work presented here are those cell-based models which consider, for example, generic epithelial monolayers. In particular, Galle *et al.* (2005) [25] present a 3D overlapping spheres model to examine growth regulation in epithelial layers, where deformation of the cells is calculated using the Hertz force law. This model considers the role of anoikis and density-dependent inhibition of cell division, and how failure of the former can be prevented from corrupting the monolayer if contact-mediated growth inhibition is applied and there is sufficiently strong cell-substrate anchorage. Schaller and Meyer-Hermann (2005) [26] propose a 3D model to investigate the growth of tumour spheroids, and while cell shapes are again defined as deformable spheres, the neighbour interactions are instead determined by a weighted Delaunay triangulation between cell centres. The dual Voronoi tessellation is applied to provide a more realistic definition of the contact surface between neighbouring cells, which is subsequently used throughout the calculations instead of the sphere contact surface. Drasdo *et al.* (2007) [27] also consider the growth of monolayers on a substrate and multi-cellular spheroids, and revisit single-layered tissues such as the blastula during development (considered in [21]) to examine the mechanical influence of contact inhibition on the growth of the cell population. Such examples demonstrate the usefulness of individual-based models to investigate the growth dynamics of epithelial cell populations.

Dunn *et al.* (2011) [28] define a discrete off-lattice cell centre multiscale model that focuses on the role of the basement membrane beneath a growing epithelial monolayer in a simplified 2D geometry: a single layer of proliferating epithelial cells constrained to lie on a rectangular bed of stromal cells, which approximate the connective tissue. Spatial connectivity is determined by a Delaunay triangulation of cell centres, and interactive forces are modelled as springs that act along the edges of this triangulation. An additional force is applied to model the role of the basement membrane, which acts in proportion to the local curvature of the epithelial layer, and to maintain a zero spontaneous curvature.

Results from this simple geometry show that a large enough basement membrane force successfully maintains a stable, flat monolayer throughout successive division events, and that increasing the strength of this force favours horizontal migration along the layer, reducing the incidence of epithelial cell detachment from the layer (whereupon cells are removed by anoikis). This work presents the foundation of a realistic representation of epithelial cell growth and migration in a deformable environment, and is extended here to model a specific case in a realistic 2D geometry – the cross section of the crypt.

Given the coupling that exists between events at the genetic level and the tissue level, it is necessary to extend the scope of theoretical modelling to address both the role of the crypt geometry and subcellular events. In addition, such a multiscale model should include a mechanical description of migration, cell-cell and cell-matrix adhesion; in so doing, the model can more fully describe all of the processes inherent in crypt dynamics and homeostasis.

The remainder of this paper is composed as follows. Firstly, experimental results are discussed that examine the tissue structure of the crypt. These results identify the composition of the connective tissue and surrounding musculature, and how the components relate to crypt shape and function. These findings are incorporated into a new crypt model which assumes the basement membrane force proposed by Dunn *et al.* (2011) [28], and investigations are conducted firstly using a simple rectangular geometry, to determine appropriate parameter balances and investigate the migration of epithelial cells out from the crypt base region. Conclusions from this modelling step inform parameter choices for a complete 2D cross-sectional geometry which is subsequently defined, and the behaviour of the extended model is demonstrated. The results and future work are discussed, where the advantages as well as the restrictions of the model are highlighted, and experiments to investigate model hypotheses are suggested. The direction for future work is outlined, centred on an extension of the 2D cross-sectional model to a realistic 3D geometry.

## Results

This section concerns experimental results that identify and describe the tissue structure of the crypts, and the results of *in silico* investigations of the theoretical model defined in accordance with this biological information. The model is constructed in two stages: firstly, by using a rectangular geometry to investigate growth of the epithelial monolayer and migration of the epithelial cells. The simple geometry is used to determine the necessary model components to achieve cell migration in the epithelial monolayer. The parameter balances determined by *in silico* experiment are then applied to a 2D cross-sectional crypt geometry. The mathematical details of the model are provided in the Materials and Methods section.

### The Tissue Structure of the Crypt

**Figure 2:**
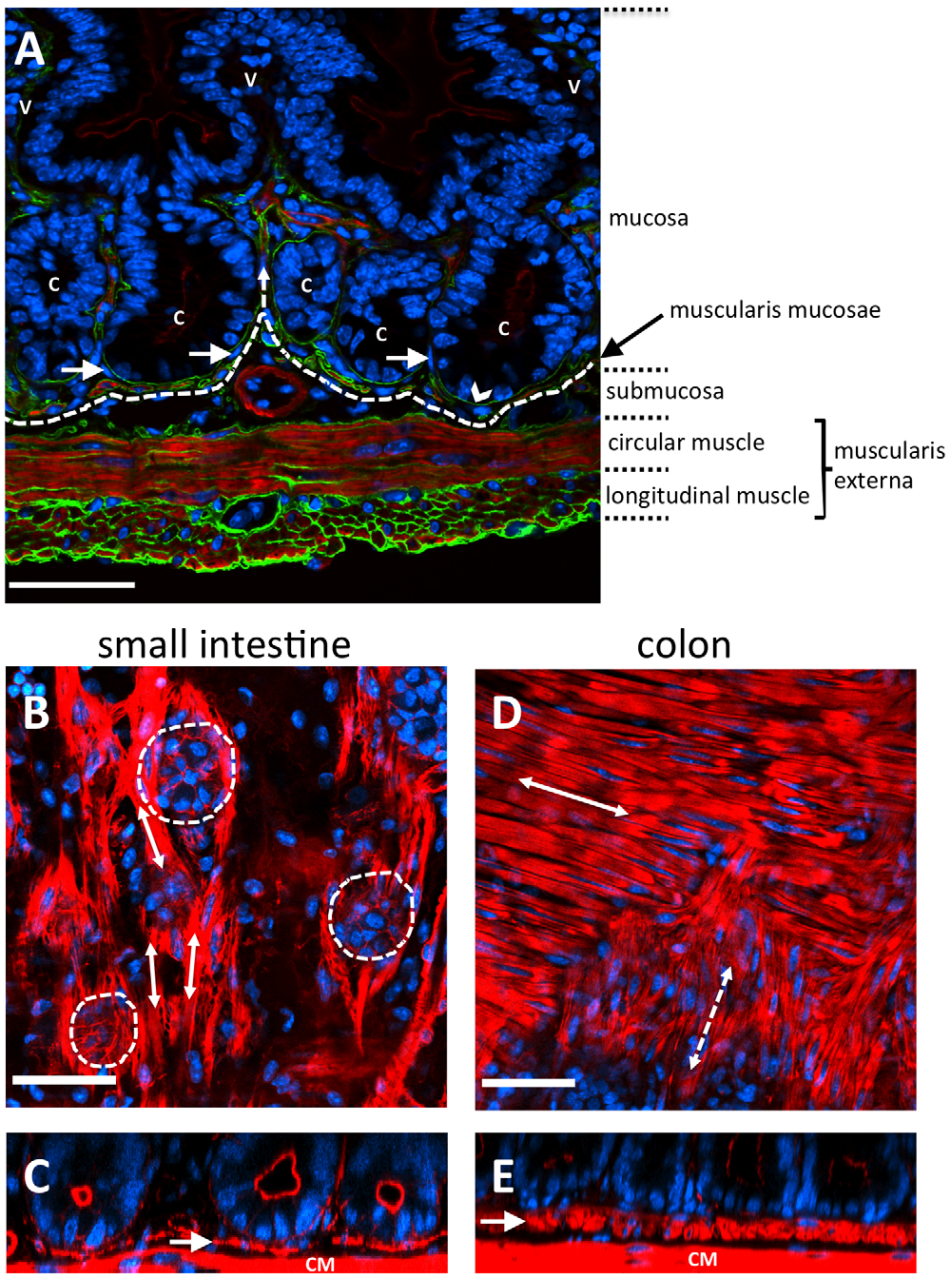
Musculature of the mouse intestinal wall. (A) A frozen section of fluorescently labelled small intestine visualised by widefield fluorescence microscopy (red shows F-actin, green shows laminin, blue shows nuclei). (B–E) Fluorescently stained wholemounts of small and large intestine, visualised using confocal fluorescence microscopy (red shows F-actin, blue shows nuclei). (A) A section through the wall of the small intestine. Nuclei (blue) are stained with DAPI, F-actin (red) highlights the smooth muscle cells and apical surface of the gut epithelium. The gut epithelium is continuous from the crypts (C) and over the villi (V). Basement membranes (green) are stained with an anti-laminin antibody, and the basal surfaces of gut epithelial cells are directly attached to the basement membrane (arrowhead). The muscularis externa is composed of outer longitudinal and inner circularly oriented smooth muscle fibres. The muscularis mucosae (just above the dashed line) closely follows the crypt bases, with some fibres extending up into the villi (dashed arrow). Pericryptal fibroblasts surround the crypt epithelium (arrows). (B) A longitudinal section through the small intestine shows the base of crypts (outlined by dashed circles). Smooth muscle fibres of the muscularis mucosae are oriented parallel (arrows) to the longitudinal muscle. (C) A transverse section of small intestine shows the muscularis mucosae (arrow). It is comprised of a single cell layer that forms an incomplete layer or meshwork beneath crypts, just above the circular muscle (CM). (D) A longitudinal section through the colon shows the outer (solid arrow) and inner (dashed arrow) more disorganised layers of the muscularis mucosae. The outer layer is oriented parallel to the longitudinal muscle and the inner layer parallel with the circular muscle, as indicated by double headed arrows. (E) A transverse section through the colon shows the muscularis mucosae (arrow) at the base of the crypts, which is much thicker than that found in the small intestine. Scale bars = 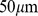.

Immediately beneath crypts lies a thin layer of smooth muscle, known as the muscularis mucosae (MM) that forms the boundary between the mucosa and submucosa, as shown in Fig. 2. In the small intestine of the mouse, the MM is one or two cells thick and forms a network that follows the contours of the crypt bases (Figs. 1 and 2). By examining intact mouse tissue in three dimensions, we found that, contrary to the reported structure of human gut tissue, the smooth muscle cells of the small intestinal MM are oriented mostly parallel to the longitudinal muscle layer of the muscularis externa (ME) (Fig. 2(A), (B), (C)). In the small intestine, the smooth muscle fibres of the MM extend up into the villi. It is thought that the role of the MM is to constantly agitate the epithelium gently to help expel secretions from crypts and enhance contact between epithelium and luminal contents [29]. When viewed in transverse section (Fig. 2(A)), the MM appears to follow closely the outline of the base of each crypt. When viewed in longitudinal section (Fig. 2(B)) the MM appears to form individual baskets beneath each crypt, analogous to an eggbox that contains each crypt base as a single egg. The MM of the colon is composed of two distinct layers of smooth muscle fibres, the outer orientated parallel with the longitudinal ME, the inner layer more disorganized, but generally oriented parallel with the circular ME (Fig. 2(D), (E)).

Other components of the mucosa are a laminin-rich basement membrane that is directly attached to the basal surface of gut epithelial cells (Fig. 2(A)) and, just below, surrounding each crypt, a pericryptal fibroblast sheath (PCFS), comprising a highly organized system of fibroblasts, collagen and mucopolysaccharide ground substance [30]. There are 38 PCFS cells per mouse small intestinal crypt and 124 per colonic crypt [31]. PCFS cells produce signaling factors involved in the growth and maintenance of the crypt. Beneath the MM lies the submucosa (SM), which consists of loose connective tissue rich in collagen and elastic fibres. Embedded in this material are larger blood vessels, lymphatics and nerves. The SM is enclosed by the muscular wall of the gut, called the muscularis. It consists of outer longitudinal and inner circular layers of smooth muscle. The muscularis is responsible for peristalsis, the contractile movements involved in advancing intestinal contents.

### Model Description

A discrete off-lattice cell centre model is defined, in which spatial connectivity is determined by a Delaunay triangulation of cell centres, and cell shapes are prescribed by the Voronoi tessellation of these centres. Interactive forces are modelled as springs that act along the edges of this triangulation, as described in the Materials and Methods section. Individual model components are now summarised. All parameters are given in Table 1.

**Table 1:** Model parameters.

Parameter	Description	Value	Units	Ref.
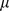	Spring strength	15	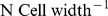	[16]
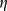	Constant drag coefficient	1	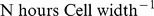	[16]
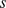	Equilibrium spring rest length	1	Cell width	[15]
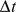	Timestep	0.0042	Hours	
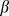	Basement membrane force parameter	see text	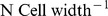	[28]
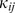	Local discrete curvature	see text	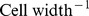	
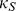	Spontaneous curvature	see text	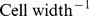	
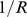	Non-zero spontaneous curvature for the crypt base	see text	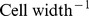	
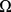	The region that defines the crypt base	see text	Cell width	
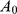	Equilibrium area of a cell	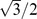	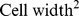	
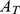	Threshold area required for cell division	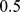	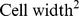	

Unless stated otherwise, the parameters in the model assume the values given in this table. The distances in the model have been scaled with cell width (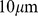 [35], [39]), time is measured in hours, and all other variables have been scaled so that 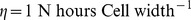 (see Equation (2)) [15], [16].

#### Geometry

**Figure 3:**
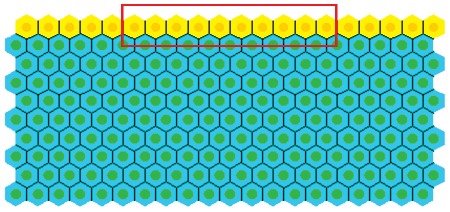
The simple geometry used to investigate the basement membrane force defined by **Equation (8)**. The top row of yellow cells are the proliferating epithelial cells, whilst the green cells are the non-proliferating stromal cells. The region of the basement membrane highlighted by the red rectangle is assigned a non-zero spontaneous curvature, 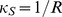, whilst the remainder outside this region is assigned a zero spontaneous curvature.

Initial model development is conducted using a simple geometry, as shown in Fig. 3. This is a 10×20 rectangular array of cells, where the top row comprises proliferating epithelial cells (yellow), and those below are non-proliferating stromal cells (green). The latter approximate the connective tissue comprising the MM, in particular the PCFS cells, and it is possible to alter the structural properties of this collection of cells by varying the interactive cell forces, which in turn define the rigidity and density. From now on, reference to the stromal cells indicates the block of connective tissue to which the epithelial cells are attached. Periodic boundary conditions are imposed on the vertical walls of the block to represent a continuous epithelial layer, and the bottom row of stromal cells are held pinned, to model the presence of the muscularis externa that sits beneath the MM (shown in Fig. 2A). Otherwise, elements of the submucosa are not considered here. The number of layers of stromal cells is chosen to allow for deformation that will occur as the epithelial layer bends downwards. The rectangular geometry is chosen to inform understanding of the basement membrane force, and is not yet intended to replicate the crypt geometry itself.

#### The basement membrane

The structural stability afforded to the epithelial monolayer by the basement membrane and PCFS is modelled using a force (subsequently referred to as the basement membrane force) that acts maintain a pre-specified spontaneous curvature. While Dunn *et al.* (2011) [28] consider a zero spontaneous curvature for this force – to model a uniformly flat, growing epithelium – the definition is extended here to define a non-zero spontaneous curvature region in the centre of the epithelial monolayer, equal to half the length of the layer (as indicated by the red rectangle in Fig. 3). This models the portion of the basement membrane and tissue stroma that sits at the base of the crypt, motivated by the observation, shown in Fig. 2, that the MM closely follows the contours of the individual crypt bases. Outside this region, the basement membrane is still assigned a spontaneous curvature of zero.

#### Cell division and death

For the simple, rectangular geometry, the epithelial cells are assigned unlimited proliferative capacity to consider a continuously growing monolayer. These proliferative cells divide stochastically, with the duration of the cell cycle (in hours) drawn for individual cells from a 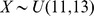 distribution. The mechanism of cell division is described in the Materials and Methods section.

Cell death via anoikis is determined using the Delaunay triangulation, which indicates whether any particular epithelial cell has lost all connections to the stromal cells, and therefore to the basement membrane (as described in [28]). In this manner, the implementation of anoikis is similar to that defined by Galle *et al.* [25], where anoikis is dependent on the contact area between epithelial cells and the substrate below, and cells are removed once this contact area reaches zero.

#### Computation

Model development is carried out within the Chaste (Cancer, Heart And Soft Tissue Environment) framework, an open source software library designed to model multiscale problems in biology, which can be accessed from http://www.cs.ox.ac.uk/chaste [32].

Unless stated otherwise, each simulation is run up to a time of 60 cell hours ( *i.e.* on the timescale of the cell cycle, not the timescale of computational simulation run time), beyond which the epithelial layer has reached a state of dynamic equilibrium and does not deform further. A typical simulation of this length requires approximately 3.7 minutes of CPU time on a desktop Linux PC (u.c.) with an Athlon 5200B processor. The code that is used to run these simulations is released and thus available to download from the Chaste website.

### Simulation Results

**Figure 4:**
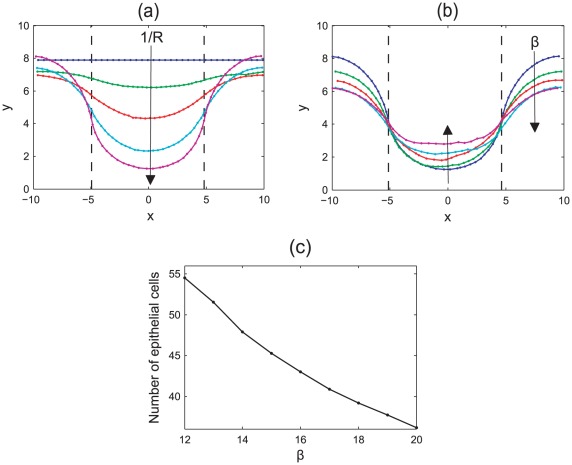
The evolution of the epithelial layer, where the 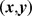-coordinates of each epithelial cell are plotted after 60 hours, and the boundaries between the zero and non-zero spontaneous curvature regions are indicated by the dashed lines. (a) 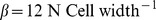, increasing 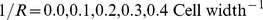 (indicated by the arrow); (b) 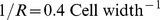 for increasing 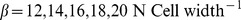 (indicated by the arrows); (c) the total number of epithelial cells in the monolayer for 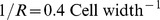 and increasing 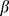.

Firstly, *in silico* experiments were run to demonstrate the effect of increasing the spontaneous curvature in the central region, 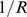, and the strength of the basement membrane force as governed by the parameter 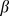, which characterises the strength of adhesion of the epithelial layer to the basement membrane and the stiffness of the membrane itself. Figs. 4(a) and (b) illustrate the change in behaviour of the monolayer by plotting the 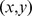-coordinates of all epithelial cells at the final timestep for typical simulations. In (a), the arrows on these plots indicate the direction of increasing 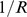, while in (b) the arrows indicate increasing 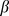. Also marked are the boundaries between the non-zero and zero target curvature regions. As these plots are generated from typical simulations, the curves are not symmetric due to recent division events.

**Figure 5:**
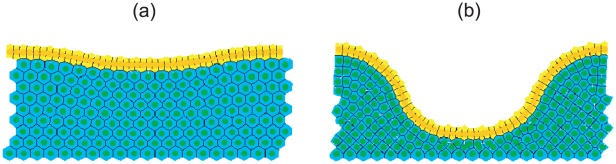
Simulation snapshots illustrating the deformation of the epithelial monolayer for increasing 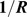. (a) 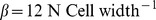, 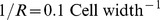; (b) 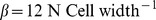, 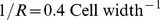. The layers have deformed from the initial state shown in Fig. 3, and snapshots are taken after 60 cell hours.

Simulations reveal that as the spontaneous curvature increases, the epithelial monolayer is pushed further down into the tissue stroma as the central portion of the monolayer bends, behaviour that is demonstrated clearly in Fig. 4(a), where 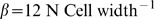. It is also observed that increasing 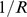 decreases the radius of the circle that can be extrapolated from the arc length of the layer – this is as expected. Simulation snapshots are shown in Fig. 5, taken after 60 hours, to illustrate the difference in deformation of the layer for 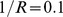 and 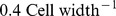.

As the basement membrane force increases, a stronger force acts on the outer edges to maintain a zero curvature, preventing these regions from bending to compensate the deformation of the region of non-zero curvature. This is emphasised in Fig. 4(b), which fixes 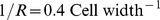. This plot shows that as 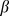 increases, the outer edges flatten and are pushed further down into the stroma. Accordingly, there is less distinction with the crypt base region, and the central portion of monolayer is not pushed down as much.

Fig. 4(c) plots the total number of epithelial cells in the layer at the final timestep for 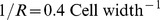 and increasing 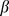, averaged over fifty simulations. This reveals the trend that the number of epithelial cells in the layer decreases as 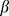 increases. As seen in Fig. 4(b), as 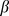 increases the deformation of the epithelial layer decreases. Correspondingly, the arc length of the layer decreases and fewer cells are held within the monolayer. To relate this to the biology of the layer, it is necessary to know more about variability in the rigidity of, and adhesion of epithelial cells to, the basement membrane.

#### Increasing cell-cell interaction forces

**Figure 6:**
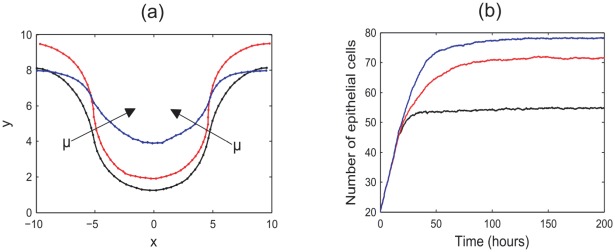
Increasing the spring constant 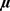 between epithelial cells. (a) Plotting the 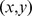-coordinates of the epithelial cells after 60 hours, where the arrow indicates the direction of increasing 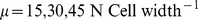. (b) Plotting the number of epithelial cells in the layer over time, averaged over 50 simulations. In both plots 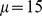 (black), 30 (red), and 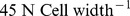 (blue).

The behaviour of the model is also influenced by the strength of the interactive cell-cell forces, which combine with the basement membrane force to determine overall cell movement. Thus, the balance of these forces is of interest. Fig. 6 illustrates the effect of varying the interactive cell-cell forces, by increasing the ratio of the spring constant between epithelial-epithelial connections and stromal-stromal connections (E-E/S-S) for 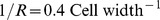 and 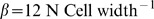. As shown in Fig. 6(a), which plots the epithelial cell locations for typical simulations after 60 hours, increasing the spring strength between epithelial cells demonstrably reduces the extent to which the layer is deformed. This occurs because the cell-cell forces dominate over the basement membrane force, preventing full deformation to the spontaneous curvature. Further, the number of epithelial cells within the layer (which increases as the layer deforms from the initial flat state until reaching a steady state) is higher for larger 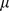. This is shown in Fig. 6(b), which plots the number of epithelial cells at each time step for increasing 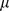, averaged over 50 simulations. This arises as a consequence of the reduced curvature of the layer, which in turn causes fewer anoikis events. The larger the curvature of the layer, the more vulnerable epithelial cells are to popping out of the layer, and so this effect is countered by the dominant cell-cell interaction forces.

#### Anoikis and cell migration

It is crucial that the application of the basement membrane force does not inhibit migration, nor induce excessive cell compression that forces cells immediately out of the monolayer following a cell division event, to be removed by anoikis. This is to ensure that the model reproduces the migratory behaviour known to occur in the crypt, in the absence of an explicitly known active migration force [33]. The following results consider and justify extensions to the current, incomplete model to generate realistic patterns of cell movement.

To examine the frequency and spatial distribution of anoikis events, 100 simulations of 500 hours of cell time were recorded from the point at which the layer had deformed to a steady state, and the results averaged. For these simulations, 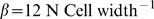, 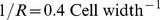.

**Figure 7:**
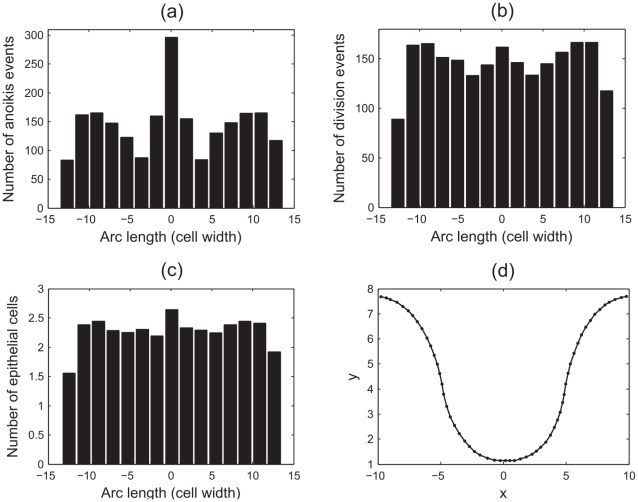
The spatial distribution of anoikis and division events, and epithelial cell locations. Here 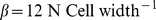, 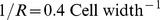, and anoikis is the only mechanism of cell removal. (a) Anoikis events, (b) division events, (c) epithelial cell locations at the final timestep, (d) a plot of the 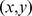-coordinates of epithelial cells in the layer at the final timestep. Arc length represents the distance along the curve of epithelial cell centres, measured from the centre of the layer.

Fig. 7(a) plots the spatial distribution of the anoikis events, where the frequency of events is plotted on the 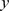-axis. The 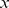-axis marks the arc length along the epithelial layer, measured from centre of the layer and calculated as the distance from this point along the curve formed by the interpolation of the epithelial cell centre coordinates. There is a clear peak in this distribution, which coincides with the centre of the curved base, where the number of anoikis events is more than double that from other regions of the monolayer. For Figs. 7(a)–(c), the edge bins show a drop in frequency that is due to the bin size itself, which is larger than is strictly necessary for these regions, as only a small portion of the monolayer extends beyond the penultimate edge bins.

Fig. 7(b) shows the corresponding spatial distribution of division events. This distribution is more uniform, with a slight increase towards the centre of the layer, and at the boundaries between the regions of zero and non-zero curvature. Similarly, the spatial distribution of epithelial cells at the end of each simulation, shown in Fig. 7(c), is fairly uniform with a slight increase in the same regions (note that this represents a snapshot in time at the end of each simulation). A typical plot of the epithelial cell centres at the final timestep is shown in Fig. 7(d).

The distribution of anoikis events does not correlate exactly with either the distribution of division events, nor the spatial distribution of epithelial cells. In particular, the sharp peak in anoikis events at the centre of the layer is much higher than the peak in epithelial cell density or cell division at this point would suggest. It is claimed here that the high number of anoikis events is a consequence of the high compression of cells in the curved region, which forces a perpendicular alignment of two neighbouring epithelial cell centres, such that one cell centre is positioned towards the lumen, and the other towards the basement membrane. Due to the Delaunay triangulation, the cell centre that moves out towards the lumen retains connections only to other epithelial cells, and not to the basement membrane, so it is removed by anoikis. It is likely that this effect is prevalent immediately following mitosis, where the two daughter cell centres are joined by a much shorter spring.

That anoikis is correlated with cell compression is in agreement with the behaviour shown in the 3D individual-based model of an epithelial monolayer proposed by Galle *et al.* [25], where it is also found that if proliferation is uninhibited, epithelial cells are forced out of the layer. Such results are common despite the difference in the models, and especially in the underlying force law (the spring law vs. the Hertz law).

The number of anoikis events also increases towards the boundary of the non-zero and zero spontaneous curvature regions, where the layer adopts a convex shape. These results suggest that the shape that the layer adopts around this boundary again renders epithelial cells vulnerable to popping out of the layer, leading to anoikis. Cells elsewhere in the layer are under lower compression, and the curvature of the layer is closer to zero, so cells are more likely to remain in the monolayer following a division event.

**Figure 8:**
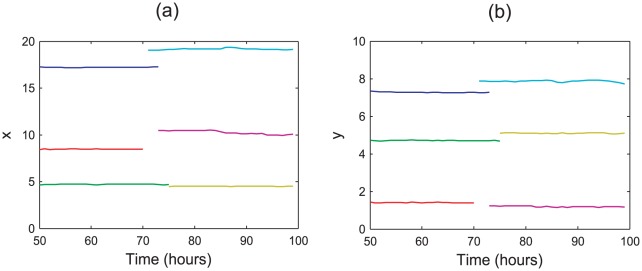
Typical cell migration in the epithelial layer when anoikis is the only mechanism of cell removal. Each colour corresponds to a different cell and tracks the 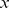 and 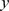-coordinates at each hour. In each case, the cells maintain a roughly constant position, showing that they do not migrate upwards: (a) 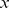-coordinates, (b) 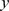-coordinates. Results are shown from 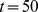 hours to allow the layer sufficient time to equilibrate.

The high incidence of anoikis events suggests that there is little, if any migration occurring in the epithelial layer. This is confirmed by visualising simulations, and also by plotting the coordinates of the cells over time. Fig. 8 plots six typical examples, each marked in a different colour, chosen to illustrate the movement that occurs at the lowest point on the monolayer, and further up the 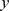-axis towards the highest point (see Fig. 7(d)). It is evident that, in each case, the cells do not migrate in either the 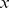 or 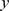 direction. Rather, the cells maintain a constant position in the layer, and are only removed due to an anoikis event. This emphasises that migration is not occurring in the model, but rather the cells have reached a state of stagnation due to excess compression and when a cell divides, it will typically undergo anoikis. This is not in agreement with known crypt dynamics, as it is established that upward migration occurs towards the crypt collar and anoikis events do not occur with such frequency at the crypt base [34]. Hence, these results suggest that additional features of crypt dynamics must also be included in the model to achieve realistic patterns of cell movement.

#### Density-dependent inhibition of mitosis

The phenomenon of density-dependent inhibition of cell division is well established, and arises in monolayers that reach confluence as a consequence of the limited availability of mitogens, growth and survival factors, preventing cells in tissues from dividing beyond a specific population density [35]. Under normal circumstances this prevents over-proliferation of cells, and experimental results indicate that growth arrest is actively induced in the G0/G1 phase of the cell cycle [36]. Thus far, the effect of density-dependent inhibition of mitosis has not been included in this model. However, the results shown in Figs. 7 and 8 indicate that when it is neglected, the correct migration pattern is not observed as cells divide despite excessive compression, and the newly created cells are forced out of the layer and removed from the simulation too quickly.

**Figure 9:**
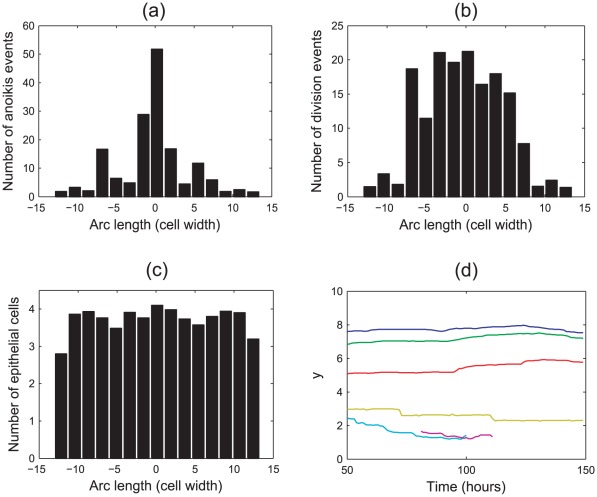
The spatial distribution of anoikis and division events that occur in the epithelial layer when density-dependent inhibition of mitosis is implemented. (a) Anoikis events, (b) division events, (c) epithelial cell locations at the final timestep, (d) six typical migratory tracks for epithelial cells in the monolayer (showing the 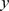-coordinates only).

To model density-dependent inhibition of mitosis, a threshold area is defined below which a cell cannot divide. Specifically, should a cell have an area, 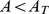, where 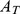 is the minimum area at which cell division can occur, then this cell will remain in G1 phase until its area grows to exceed this threshold. In these simulations, the equilibrium area for each cell is 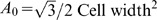, and 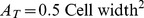. Again, 100 simulations were run, each for 500 hours of cell time, with 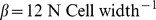, 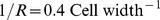. The results are shown in Fig. 9.

Fig. 9(a) shows that the spatial distribution of anoikis events still peaks at the centre of the monolayer. Comparison with Fig. 7(a) reveals that while the frequency of anoikis events has reduced (again, this is in agreement with the model proposed by Galle *et al.* [25]), the proportion of events happening at the centre of the monolayer has increased. Fig. 9(b) shows that the pattern of cell division has changed, with fewer division events occurring towards the edges of the monolayer (where there are fewer cells), and the overall frequency of division events is greatly reduced.

The distribution of epithelial cells at the end of the simulation is fairly uniform, as shown in Fig. 9(c), and is unchanged from that presented in Fig. 7(c).

Fig. 9(d) tracks the 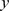-coordinates of six cells, displaying typical behaviour for epithelial cells at different positions in the monolayer. As in Fig. 8, the epithelial cells are not migrating up the monolayer, but remain in approximately the same vertical position. Compared to cells further up the 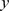-axis, those cells closest to the lowest point of the monolayer typically live for a shorter period before being removed by anoikis.

These results show that, with density-dependent inhibition of mitosis, cells are prevented from dividing when under compression so that once the layer has reached a state of equilibrium, migration does not occur as too few cells are introduced to the simulation. Thus, the model does not yet accurately reproduce the known dynamics of cells within the crypt and at this stage, is incomplete. It is necessary to include a further, known component of crypt cell dynamics: cell death at the crypt collar.

#### Cell death at the domain boundaries

**Figure 10:**
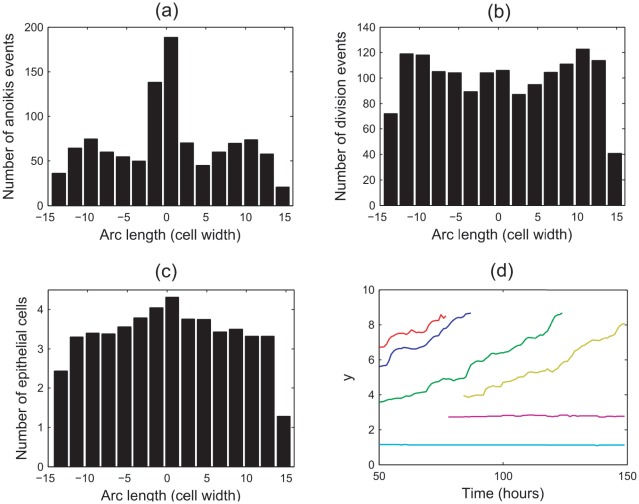
The spatial distribution of anoikis and division events that occur in the epithelial layer when density-dependent inhibition of mitosis and random cell death are implemented. (a) Anoikis events, (b) division events, (c) epithelial cell locations at the final timestep, (d) six typical migratory tracks for epithelial cells in the monolayer (showing the 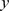-coordinates only).

Experimentally, it has been found that as the cells in individual crypts migrate upwards to the intercrypt table, apoptosis/sloughing occurs at the crypt collar. With no mechanism of cell loss in the current model other than anoikis, the results shown already indicate that cells are prevented from migrating and dividing as the layer is overly compressed. Thus, to consider the effect of a second mechanism of cell loss known to occur in reality, random apoptosis events are now defined to occur in the outer portions of the epithelial layer. Specifically, those cells in the regions 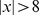 have a probability 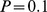 of starting apoptosis within each hour of simulation. Again, 100 simulations were run, each for 500 hours of cell time, with 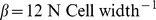, 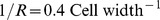. The results are shown in Fig. 10, and the frequencies presented correspond to an average over all 100 simulations.

Fig. 10(a) plots the spatial distribution of anoikis events under these conditions, and Fig. 10(b) plots the spatial distribution of division events. When compared with Fig. 7 there is a reduction in both the number of anoikis and division events, but these frequencies are higher than when density-dependent inhibition of mitosis is implemented solely with anoikis.

Fig. 10(b) shows that the number of cell births increases moving out from the non-zero spontaneous curvature region towards the zero spontaneous curvature region, and this distribution of division events has changed from when only anoikis and density-dependent inhibition of mitosis were implemented. This highlights the fact that the cells at the crypt base are still compressed, but there is migration occurring in the layer which allows the epithelial cells to achieve a sufficiently large area to undergo cell division. Moreover, the spatial range of anoikis and division events, as well as the spatial range of epithelial cells as shown in Fig. 10(d), has extended, which reveals that the monolayer has grown beyond the length observed in Figs. 7 and 9. There is also a change in the distribution of epithelial cell density at the final timestep, which now decreases moving outwards from the centre of the monolayer.

**Figure 11:**
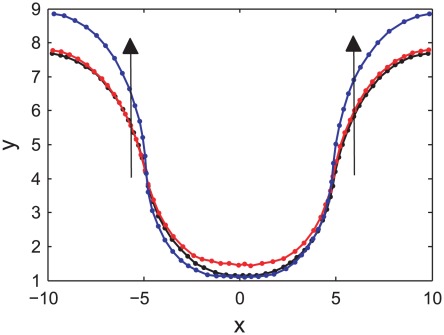
A comparison of the final state of the epithelial layer for the three cases considered. The direction of the arrows indicate the extension of the epithelial layer when random cell death is included at the edges (blue curve), compared to the two cases where this second form of apoptosis is not present.

The growth of the epithelial monolayer is illustrated in Fig. 11, which compares the typical final state of the epithelial layer for the three cases considered here: (i) anoikis only, (ii) anoikis and density-dependent inhibition of mitosis, and (iii) anoikis, density-dependent inhibition of mitosis and random cell death imposed at the edges of the monolayer. The arrows in the plot indicate that for the final case, the epithelial monolayer has extended and grown upwards.

These results are confirmed by examining the migratory patterns of epithelial cells. Fig. 10(d) plots the 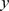-coordinates of six typical epithelial cells over time, and reveals two distinct types of behaviour. Firstly, those cells sitting at the lowest point of the monolayer tend to remain in position and do not migrate upwards. In contrast, those cells further up are shown to migrate upwards, before being removed either by anoikis, or by the random apoptosis at the edges. This is in agreement with the known cell dynamics of the crypt – stem cells that sit at the base of the crypt do not migrate upwards, but rather maintain a steady population through asymmetric division events, despite not being fixed in position (as was the case in the model due to Meineke *et al.* [16]). However, both transit-amplifying cells and differentiated cells migrate towards the crypt collar.

Therefore, including apoptosis towards the edges of the epithelial monolayer, as occurs in the crypt geometry itself, increases the rate of cell migration by establishing a feedback mechanism whereby apoptosis creates additional space for neighbouring epithelial cells to move into, allowing the cells to equilibrate and then to divide – this is similar to the negative pressure hypothesis [37]. From these results, it is concluded that for cell migration to occur in the epithelial monolayer, it is necessary to include a second mechanism of cell removal at the top of the crypt, in addition to density-dependent inhibition of cell division.

#### Conclusions

As shown in Fig. 2, the MM appears to follow closely the outline of the base of each crypt, forming individual supports for each crypt, analogous to an eggbox that contains each base as a single egg. This motivates the definition of a non-zero spontaneous curvature region of the basement membrane beneath the crypt base, and the results shown thus far reveal that under this assumption, the application of the basement membrane force has the desired effect of deforming the layer to adopt a semi-spherical shape. To ensure that the radius of the crypt base region is not too large, a high spontaneous curvature should be chosen, but then to prevent excess deformation of the stromal tissue below, the ratio of the E-E/S-S spring strengths should also be high. To prevent the horizontal, *i.e.* zero spontaneous curvature, portions of the layer from being pulled down, the minimum effective value of 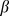 should be chosen.

Simulation results have also revealed insight into a possible mechanism behind the migration of cells in the epithelial monolayer. Currently it is assumed in biology that there is some form of active migration in the crypts, but it is not known what may drive this force [38]. *In silico* simulations of the theoretical model proposed here have shown that to ensure that cell migration occurs, cell removal must be implemented to mimic the apoptosis/shedding that occurs at the crypt collar. This is in addition to density-dependent inhibition of cell division, which prevents the overproduction of cells in compressed regions. Hence these results indicate that the cause of migration in the epithelial layer may not be solely due to an active force that pushes cells up the crypt, but that a feedback mechanism may exist between cell birth and cell death, such that apoptosis at the crypt collar creates additional space for epithelial cells to move into. This subsequently relieves the compression in the monolayer, allowing epithelial cells to grow sufficiently to progress through the cell cycle and divide, propelling migration and maintaining barrier function. This is a plausible hypothesis generated via *in silico* experiments, which is testable in a wet lab. A typical simulation example of the model in the current form is provided in supplementary video S1.

### Modelling the Cross-Section of a Crypt

**Figure 12:**
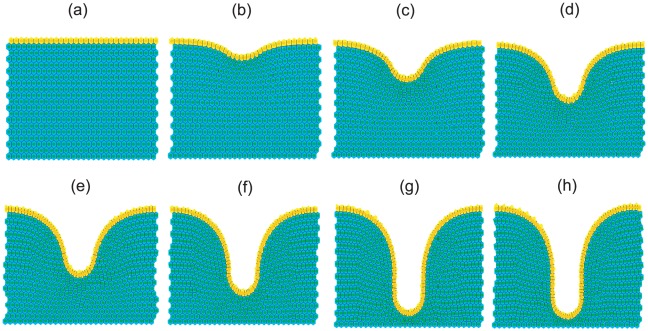
Deforming a flat epithelial layer to adopt the test-tube shape of the crypt, as viewed in a cross-section. To achieve this, a non-zero spontaneous curvature region is defined in the centre of the monolayer, equal to 20% of the width of the tissue block. It is also necessary to increase the size of the stromal cell compartment, as well as define a sufficiently wide epithelial monolayer. Each image from (a) to (h) is a snapshot in time, and the layer is fully deformed after approximately 100 cell hours.

The simple geometric framework employed for the investigations thus far is a limiting factor preventing realistic modelling of the colonic epithelium, and so the next step is to incorporate the crypt geometry. As shown in Fig. 12, it is possible to deform an initially flat epithelial monolayer to adopt a test-tube crypt shape by suitable application of the basement membrane force (see supplementary video S2). However, in order to do so it is necessary to define the initial rectangular geometry to be sufficiently wide, which in turn increases the width of the tissue stroma surrounding the crypt once the layer has fully deformed. This is unrealistic, as the stroma between neighbouring crypts is only 2–3 cells thick. The starting point for the following simulations is instead an initial geometry that approximates the shape of the crypt, described below. Distinct proliferative compartments can be defined as dependent on an imposed Wnt gradient, and this also has the advantage of eliminating the time required to fully deform the flat layer. From the approximate geometry, the basement membrane force acts to maintain the test-tube shape within the tissue through local calculation of the discrete curvature. This is a key feature of the model, as the test-tube geometry emerges due to the action of the forces, rather than being fixed and imposed as in most earlier models.

The results found for the simple rectangular geometry are now translated to the cross-sectional geometry. The conditions required for homeostasis are sought, which present a balance between the basement membrane force and the adhesion and repulsion between neighbouring cells, to allow constant upward migration that is matched by cell removal at the collar. Thus, the number of epithelial cells in the crypt should fluctuate only slightly around a constant value, the cells should not be overly compressed, and the structure should not buckle.

#### Geometry

**Figure 13:**
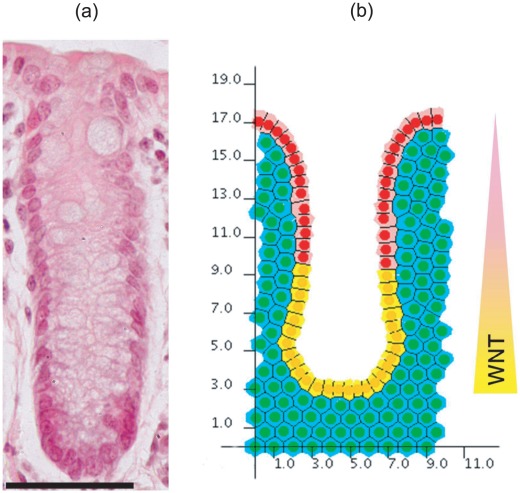
The cross-sectional crypt configuration. (a) A haematoxylin and eosin stained wax section of a murine colonic crypt. Scale bar = 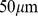. (b) The cross-sectional model geometry, plotted against the axes used to mark cell 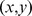-coordinates for the results presented. A linearly decreasing gradient of Wnt factors is imposed in the cross-sectional model, which is normalised to 1 at the base of the crypt, and 0 at the crypt collar. A threshold is prescribed such that cells in the region of insufficient Wnt factors are defined to be terminally differentiated. Thus, proliferating and non-proliferating epithelial cells are indicated in yellow and pink respectively, whilst non-proliferating stromal cells are indicated in green.

The new geometry is defined to represent a slice taken longitudinally from the apex of the crypt to the collar and is shown in Fig. 13, which places the model in the context of a histological slice of a crypt. The epithelial layer of the crypt is thus a 1D chain of cells embedded in a 2D rectangle of stromal cells that represent the connective tissue. Wnt-dependent proliferation is imposed to distinguish between non-proliferating epithelial cells, which model the terminally differentiated cells towards the crypt collar and are coloured pink, from the proliferating epithelial cells which are coloured yellow. This dependence is described below. Again, periodic boundary conditions are imposed on the vertical walls of the tissue block to mimic the existence of neighbouring glands on either side of the crypt. Those cells lining the base of the stroma are pinned to model the stabilising effect of the muscularis externa (see Fig. 2).

#### Cell division

Now that a realistic crypt shape is adopted, it is necessary to model the distinct proliferating and non-proliferating regions of the epithelial monolayer. To achieve this, a linearly decreasing Wnt concentration gradient is imposed along the vertical axis of the crypt, which is normalised to decay from 1 at the crypt base to 0 at the crypt collar (Fig. 13(b)). Each cell detects the Wnt level at its centre and a threshold is prescribed such that cells that are positioned in a region of sufficient Wnt proceed through the cell cycle and undergo division. Those cells that are positioned higher up the crypt experience a lower level of Wnt that is below this threshold, are so are defined to be terminally differentiated and do not divide. This models a discrete point at which epithelial cells stop proliferating in the crypt [15]. The duration of the cell cycle (in hours) is again randomly drawn for each proliferative cell from an 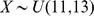 distribution and cell division is conducted as described in the Materials and Methods section.

#### Parameter choices

Starting from an approximate geometry, the basement membrane force acts to maintain the test-tube shape of the crypt within the tissue due to the local calculation of the discrete curvature. The choice of 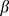 is influenced by the new geometry in light of the effect that it has at the curve of the monolayer where the vertical walls meet the horizontal intercrypt table. As the spontaneous curvature is zero everywhere save at the crypt base, the action of the basement membrane force will cause the corners to flatten, and in so doing, will lower the intercrypt table and cause the crypt to shrink in height. To prevent this having a dominant effect on the structure, a low value of 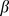 must be chosen, but one which still defines a large enough basement membrane force to keep the vertical walls of the crypt column flat. Thus it is sensible to choose 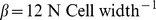. This is in agreement with the results shown in Fig. 4(b).

The results from the rectangular geometry indicate that for 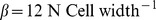, a spontaneous curvature of 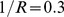 or 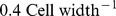 is optimal for the crypt base, to ensure a sufficiently small radius. Lastly, to prevent excess deformation of the tissue stroma, a large enough spring constant must be chosen, 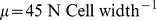. Density-dependent inhibition of cell division is implemented, and random death events occur within the top 10% of the crypt length.

**Figure 14:**
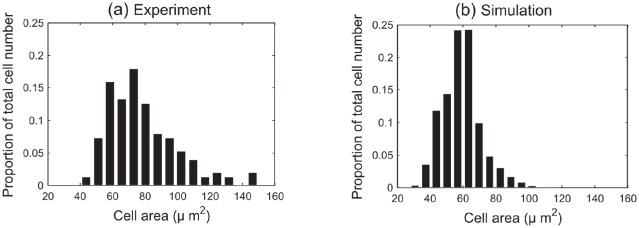
A comparison of model results with cell area data for three healthy murine colonic epithelium samples. (a) Experimental data obtained from three wildtype murine tissue samples, taken from midway down the length of the colon. (b) A histogram of cross-sectional area data collected from the simulation of 1000 hours. These data have been dimensionalised, scaling 1 cell width to 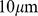, and the frequency has been averaged over total cell number to show the proportion of cells in each bin.

Parameter estimation for cell width in the model is achieved by comparison of simulation data with experimental results for three wildtype murine crypts, the details of which are provided in the Materials and Methods section. Fig. 14(a) is a histogram of cross-sectional cell area data obtained from colonic epithelium samples taken from midway down the length of the colon in three wildtype mice, where the frequency is averaged over the total cell number to indicate the proportion of cells within each bin. Despite this being a small data set, it serves as a guide to what is found realistically in the murine crypt. This histogram shows that cross-sectional cell area can range from 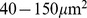, and there is a peak between 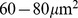.

Fig. 14(b) is a histogram of cross-sectional cell areas obtained by running 1000 hours of cell time in the cross-sectional model. By choosing a typical cell width of 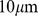 [35], [39], these histograms compare well, and the simulations demonstrate a range of approximately 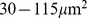. These results indicate that despite only considering a cross section of the crypt, the turnover and cell numbers in the model lead towards plausible and realistic cell areas.

#### Results

**Figure 15:**
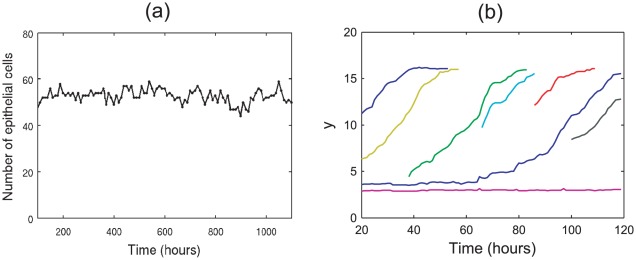
Cell turnover statistics for the cross-sectional model. (a) The total number of epithelial cells in the crypt over time. (b) Typical migration tracks for cells that are born at different positions in the epithelial monolayer (each coloured curve tracks the 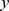-coordinate of a specific epithelial cell, each born at different locations in the crypt).

Simulations reproduce the qualitative behaviour of the crypt when the parameters chosen above are implemented: 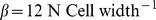, 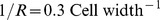 and 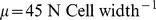. The crypt achieves a state of dynamic homeostasis with repeated mitotic events matched by cell death to achieve a steady rate of cell turnover, with cell migration directed towards the crypt collar, as illustrated in Fig. 15.

Fig. 15(a) plots the total number of epithelial cells in the crypt over time, for a typical simulation of 1000 cell hours. Cell number remains approximately constant, and fluctuates between 50–60 due to the stochasticity of cell birth and cell death in the model. (In this plot, results from the first 100 hours are omitted to ensure that equilibrium has been reached.)

The results for the rectangular geometry show that outward migration occurs from the base of the non-zero spontaneous curvature region towards the edges (Fig. 10(d)). This pattern of cell movement away from the crypt base is also found in simulations of the cross-sectional model and qualitatively matches the upward migration that is observed biologically. This is demonstrated by the typical migration tracks plotted in Fig. 15(b), which illustrate the common upward movement of epithelial cells over time. The plot also shows an example of a cell born at the base of the crypt, which tend to remain in that region, as is the case for stem cells in the crypt. It is possible, however, for such cells to migrate out of this zone and move up to the crypt collar, as shown by the blue curve in the plot.

**Figure 16:**
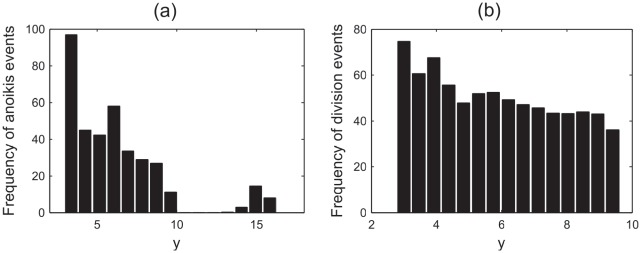
Cell division and death statistics for the cross-sectional model. (a) The spatial distribution of anoikis events along the 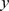-axis, (c) the spatial distribution of division events along the 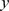-axis.

To generate a representation of the typical distribution of anoikis and division events, 50 simulations of 500 cell hours were run. Fig. 16(a) plots the spatial distribution of anoikis events along the 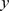-axis (where the axes used are those shown in Fig. 13(b)). It is observed that there is a correlation between the range of the proliferative compartment (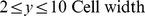) and the incidence of anoikis events. In particular, a high incidence of anoikis events occurs at the very base of the crypt, which was also found in the simple, rectangular geometry, whereas cell death is not commonly observed here experimentally [34]. The correlation suggests that, in the model, many anoikis events occur immediately following mitosis.

The process of asymmetric division, which occurs in the stem cell pool at the base of the crypt, involves a perpendicular alignment of the mitotic spindle, causing the apical displacement of one daughter nuclei, which later reinserts basally. In this model there is currently no torque force applied to pull such cell centres back into the monolayer, as biologically it is not known how this process occurs. However, these results suggest that mechanical compression of cells at the crypt base may play a role in causing asymmetric division by forcing the perpendicular alignment of the spindle. In contrast, those cells further up the crypt do not move out of the layer as often, and this correlates with the expected symmetric division for epithelial cells in the transit-amplifying compartment.

Further, it can be seen from Fig. 16(a) that, typically, anoikis events do not occur in the differentiated cell zone, save at the curve of the crypt collar (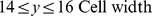). This is in agreement with the results found for the simpler geometry, and it is suggested that the crowding induced by the negative curvature at the crypt collar renders the differentiated epithelial cells vulnerable to extrusion. Biologically, cells in this region can have a reduced basal contact area, *i.e.* experience a loss of substrate adhesion, which may further contribute to these events.

Fig. 16(b) plots the spatial distribution of division events along the 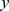-axis. In correlation with Fig. 16(a), a large number of division events occur at the crypt base, and the frequency decreases slightly moving up the vertical crypt axis (no division events occur for 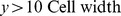). Despite this correlation, not all birth events result in anoikis, as can be observed in the simulation video S3.

## Discussion

The work described in this paper thus far constitutes the foundation of a realistic, theoretical representation of growth in a deformable environment within the colonic crypt. The usefulness and the need for mathematical modelling as a tool to guide and inform biological experimentation is becoming increasingly recognised [40]. In particular, the field of oncology lacks a comprehensive model to which existing data can be applied, nonetheless such a model is required to identify key system parameters [41]. The collaboration that resulted in this paper is intended to produce qualitative results that identify parameter balances and mechanisms that describe the behaviour of the system and which cannot be obtained by alternative methods for ethical, financial or viability reasons. Such results will inform experimental work, and identify areas for future investigation.

A simple approach is adopted to describe the evolution of the tissue structure while linking subcellular processes (Wnt signalling, cell cycle control, cell adhesion, cell differentiation) with cellular mechanics that control division, migration and apoptosis. The role of the basement membrane is defined by an additional force which takes into account the structural support provided by the surrounding connective tissue, including the PCFS. Evidence for this is based on experimental observation of the tissue structure of the crypt *in situ*, reported here, which identifies key components that contribute towards crypt shape and function.

Although only the colonic crypt is considered here, extension to consider the small intestinal crypt would be possible based on this work, but would require the definition of the villus and the incorporation of paneth cells. This is a possible direction for future work. In addition, one could examine the effect of possible gradients of adhesion along the crypt axis, via a spatially-dependent basement membrane force parameter, and monitor any subsequent change in cell migration or cell death.

Simulations of the cross-sectional crypt model demonstrate that dynamic homeostasis can be achieved, in which repeated mitotic events evolve to force consistent epithelial cell migration towards the crypt collar, without compromising the overall structure and architecture. This is characterised by a steady, constant turnover of cells, achieved in the presence of known constraints on the number of dividing cells, and applying the two known mechanisms of cell death. It is by the application of the basement membrane force that the shape of the crypt evolves, and will allow the structure to deform, rather than imposing a fixed geometry. This is a key aspect of this work, given that all previous crypt models, with the exception of Drasdo and Loeffler (2001) [21], have assumed a fixed geometry [15], [16], [18], [24], and permits investigation of the destabilisation of the crypt structure that occurs at the onset of carcinogenesis, and indeed which can aggravate the growth of a pre-cancerous adenoma.

Moreover, two insights are proposed regarding mechanisms of cell dynamics within the crypts. Firstly, in the absence of sufficient apoptosis at the crypt collar and intercrypt table, epithelial cells reach a state of confluence, do not divide and migration is inhibited. This suggests that cell migration in the crypt may not be due solely to proliferative pressure from below, but that a feedback mechanism exists between cell birth and cell death, such that the epithelial cells move into the space created by cell death at the collar. Subsequently, cells below are able to grow, divide and migrate upwards, which has the secondary effect of maintaining barrier function. This is a theory that is in line with the extrusion process that occurs for apoptotic cells in epithelial layers [42], [43] and is known as the negative pressure hypothesis [37]. In the model, apoptosis has been defined to occur randomly, given that the cause of cell removal at the crypt collar is currently unknown. It is likely that programmed cell death acts in combination with anoikis events that may be induced by the crowding of cells at the crypt collar, shown in Fig. 16(a), where the layer has a negative curvature, to remove cells and ultimately enable cell migration. To test this hypothesis in a wet lab, it is suggested that apoptosis could be induced uniformly along the crypt-villus axis, and any alteration to the typical migratory pattern subsequently monitored.

Secondly, the model demonstrates a high incidence of anoikis events at the crypt base. This is not commonly observed by experimentalists [34]. However, when these results are considered in light of the process of asymmetric division in stem cells, whereby one nuclei is positioned apically (towards the lumen) before reinserting basally by an as yet unknown mechanism, it is suggested that the crypt shape may play a role in forcing the alignment of the mitotic spindle for the compressed cells at the base. In the model, the compression of cells at the base forces one of the daughter cell centres to lose contact with the basement membrane, whereupon it is removed and this registers as an anoikis event. That a high incidence of anoikis events happen following division at the base of the crypt therefore indicates a mechanosensory cause for asymmetric division in the stem cells at the base of the crypt. This hypothesis is supported by experimental results which demonstrate that cells do respond to their mechanical environment, and moreover that cytokinesis is a mechanical process [44]. It has also been suggested that the shape of cells and tissues can influence cell division via cortical tension heterogeneity which guides spindle orientation [45].

The simulation results also show that anoikis events occur at the curve of the crypt collar. As cells in this region are now differentiated, it is hypothesised that such events arise due to the negative curvature of the layer at this point, rendering cells vulnerable to extrusion. To test this, one could grow epithelial cells on curved substrates to examine the incidence of extrusion on negatively curved regions. Alternatively, cells could initially be grown on a flat substrate, which is bent once the cells reach confluence.

The experiments suggested above identify ways in which model development and wet lab experiments can enter a feedback loop to advance understanding of the system. Support or invalidation of the hypotheses proposed will guide future model iterations, generally advancing the understanding of the system. Ideally, the most useful experiments would be those imaging live tissue using appropriate markers, so as to measure individual cell behaviours over time. This would be another way to parametrise the model out of imaging data (in addition to that used to generate the data shown in Fig. 14). It has been demonstrated that crypt-like organoids can be grown in matrigel in the presence of growth factors distributed uniformly throughout the gel, which appear similar to crypts in tissue. That additional forces do play a significant role *in vivo* is illustrated by the fact that crypts which are mutant in Apc form apparently normal shapes in whole tissue, but do not in matrigel [3]. Therefore, at this stage, it is not yet possible to state decisively how crypt organoids could be used to test hypotheses generated by theoretical models.

When constructing a mathematical model of a biological system, it is wise to keep the model as simple as possible, focussing accurately on the key components and understanding the outcome of crucial interactions without over-complicating the description and analysis required [46]. However, the computational framework within which the crypt model has been developed (Chaste) makes it extensible and amenable to additional complexity, should it be required. For example, more detailed cell cycle models can be applied, and many currently exist within the Chaste framework.

At present, however, there are limiting factors that prevent full examination of the destabilisation of the crypt structure that occurs in the progression from a healthy system to the growth of a malignant tumour. As the cross-sectional model consists of a 1D chain of cells, it is only possible for epithelial cells to move vertically or to displace the surrounding stroma. Consequently, the introduction of a mutant cell that migrates aberrantly, *e.g.* more slowly, will always affect those cells directly beneath it in the chain. In reality, these cells would be able to move around a blockage by moving laterally across the inner surface of the crypt. To correct this, it would not be sufficient to simply apply radial symmetry to the model, a method that would produce unrealistic results due to the imposed symmetry, causing mutations to spread uniformly upwards as cells migrate. In contrast, a full deformable 3D model which permits lateral movement could eliminate false positives observed in the 2D model. For example, a detailed response of the system to the introduction of mutant cell populations could be obtained, and meaningful experiments to investigate the “top-down” [47] and “bottom-up” [48], [49] theories of mutant cell invasion could be conducted. In addition, a full 3D model makes it possible to define a localised stem cell compartment within the crypt that is distinguished from the transit-amplifying cell compartment by specific proliferative properties. (In 2D, the cell chain would force stem cells out of the base of the crypt.) This would enable investigation of stem cell number in the crypt, an open question within the field, and how this affects cell dynamics.

The additional degrees of freedom associated with cell movement introduced by a deformable 3D model will increase the scope for accurately modelling the response of the system to different cellular events. For example, the merging or rearrangement of cell columns, causing the lumen to narrow or widen should the number of cells decrease or increase. This will have bearing on the persistence of mutations in the crypt, as well as on the incidence of anoikis events, which are likely to decrease as cells can exploit movement and growth in more directions. However, it is necessary to re-evaluate the structure of forces in 3D, firstly in light of the additional complexity of the Delaunay triangulation, and also to consider the effect of shear forces that may contribute to the stability of the structure. This work is underway.

## Materials and Methods

For the experiments described below, all animals were kept as governed by a valid Home Office License, but no procedures that require ethical approval were involved.

### Morphology of Gut Musculature

Mouse gut tissue was prepared for imaging following the methods described in Appleton *et al.* (2009) [50]. Sectioned gut was imaged on a Leica DMIRB fluorescent microscope and the wholemounts imaged on a Zeiss 710 confocal microscope.

### Measurement of Cell Area in Mouse Colon

3D images of fixed whole-mount tissue stained with DAPI and rhodamine-phalloidin were acquired using multiphoton fluorescence microscopy [50]. The outer surface of the crypt is defined by the basement membrane on which the epithelial cells sit, and the outer crypt area and crypt lumen area were measured in a cross section half way along the crypt length using Volocity image analysis software (Perkin Elmer). The cell area was estimated by subtracting the lumen area from the outer crypt area, followed by dividing by the number of DAPI-stained nuclei present in the center cross section. A total of 150 crypts were examined, taken from the colons of three wild type C57BL/6J mice.

### Model Components

A discrete multiscale model is considered, where cell centres are defined as nodes which evolve spatially according to an off-lattice definition of cell-cell mechanics [15], [16]. As such, spatial connectivity is determined by a Delaunay triangulation of cell centres, and the corresponding cell shapes are subsequently defined and visualised by the dual Voronoi tessellation, which has been shown to produce realistic polygonal cell shapes [51]. An example of this triangulation and tessellation is illustrated in Fig. 3.

#### Interactive cell forces

The interactive forces between cells, which correspond to cell-cell adhesion as well as the limited compressibility of each cell, are modelled here as linear springs that act along the edges of the Delaunay triangulation. The total force acting on each cell centre 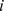 is found by calculating the sum of the contributing forces from the springs that connect it to each neighbouring cell centre, 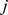:
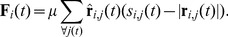
(1)Here, 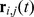 is the vector from cell centre 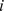 to 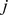, 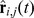 is the corresponding unit vector, 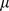 is the spring constant and 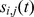 is the spring rest length, which is equal for all connections, save those between cells that have just undergone division (described below).

By neglecting inertial terms relative to dissipative terms, the velocity of node 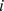 is given by
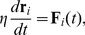
(2)where 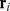 the position of node 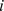, and 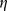 is a constant drag term, which represents general resistance analogous to motion in an aqueous medium. The system is evolved in small intervals, and cell positions are updated at each timestep using the Forward Euler method [52].

#### Cell division

The proliferative epithelial cells in the monolayer divide according to a simple stochastic cell cycle model, which assigns a G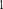 phase duration to each cell that is sampled from a Uniform 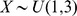 distribution. The remaining phases of the cell cycle are held constant and assigned the following durations so that the cell cycle duration for each proliferating cell is between 11–13 hours [53], [54]: G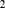 phase of 4 hours, S phase of 5 hours and M phase of 1 hour. The stromal cells are defined to be terminally differentiated, and so do not divide.

To model the directed cell division that arises from planar cell polarity, each cell is instructed to divide according to the relative position of the nearest epithelial neighbours. Two daughter nodes are created in place of the original parent node, connected by a spring that lies parallel to the vector that connects the nearest neighbours of the parent node, and placed a small distance away (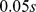) on either side of the parent node position, where 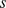 is the mature spring rest length. The new connecting spring is assigned a rest length 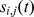, which increases linearly from 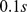 to 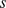 over the course of M phase (1 hour), which models the growth of a cell before division occurs, at which point two new daughter cells are created in the tessellation.

#### The basement membrane force

**Figure 17:**
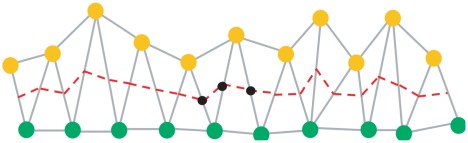
Defining the location of the basement membrane. The red dashed line indicates the location of the basement membrane, which is defined to pass through the midpoints of the springs connecting neighbouring epithelial and stromal cell centres. The local discrete curvature is calculated for each epithelial and stromal node pair, and the midpoints of the neighbouring springs are taken to form a piecewise linear curve defined by three points. An example is indicated by the three points marked by black circles.

As described and first defined in Dunn *et al.* (2011) [28], the basement membrane to which both epithelial and stromal cells are connected is modelled as a piecewise linear curve that passes through the midpoints of the springs connecting such neighbouring epithelial-stromal cell pairs, as shown in Fig. 17. Structural support is afforded to the crypt by the surrounding connective tissue and musculature, and experimental observations reveal that the MM closely follows the crypt bases, which are also surrounded by the PCFS (see Fig. 2); it is proposed here to model this support using a restorative force that is dependent on the local curvature of the basement membrane, and acts to maintain a specified spontaneous curvature that varies spatially.

The basement membrane is modelled as a piecewise linear interpolation of the spring midpoints, and so the discrete equivalent is sought for the curvature, which is hereafter referred to as the discrete curvature. The definition of curvature for a smooth, continuous curve is as follows. Let 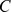 be a curve defined parametrically by 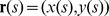, where 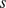 is arc length. The signed curvature, 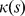, at a point 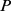 on 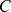 measures the rate of change, 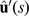, of the unit tangent vector, 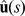, at 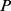. Now, 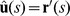 and thus
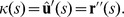
In terms of the coordinates 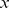 and 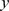, this is
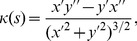
(3)where 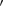 denotes 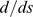.

For the purposes of this model, it is necessary to approximate Equation (3) to calculate the discrete curvature at a point 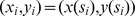 on a piecewise linear curve defined using the two neighbouring points, 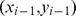 and 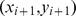. Define
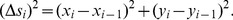
(4)The positive square root is taken so that
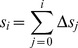
(5)is strictly increasing. The first and second derivatives are approximated using central differences [55] so that
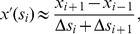
(6)

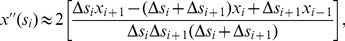
(7)and 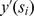 and 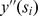 are found similarly.

These approximations are substituted into Equation (3) to calculate the discrete curvature at a particular spring midpoint. The discrete curvature is then used to calculate a restoring force acting on each epithelial node 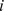 subject to each cell 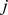 in the set of neighbouring stromal cells 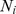:
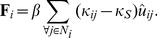
(8)This convenient form is chosen to mimic the resistance of the layer to adopting a curvature different to that prescribed by the spontaneous curvature. Here 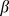 is the basement membrane force parameter, which characterises the strength of adhesion of the epithelial layer to the basement membrane and the stiffness of the membrane itself, 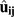 is the unit vector from the stromal node 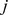 to the epithelial node 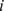, and 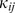 is the calculated local discrete curvature for this cell pair. The value of the spontaneous curvature, 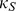, depends on where a particular epithelial cell lies, and takes the values
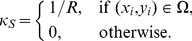
(9)Here 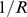 is the non-zero spontaneous curvature to be determined by numerical experiment, where 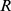 is the radius of curvature, 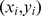 are the coordinates of cell centre 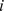, and 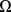 corresponds to a region defined by the crypt base. For the simple rectangular geometry, 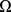 is the region enclosed by 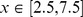, which corresponds to a region equal in length to half of the monolayer, but positioned at the centre as indicated by the red rectangle in Fig. 3. In the cross-sectional geometry, this corresponds to the crypt base region, which is defined by 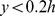, where 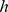 is the height of the crypt (which is calculated dynamically).

The parameters used in these simulations are stated in Table 1. Based on the results found by Dunn *et al.* (2011) [28], a value of 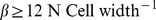 is sufficient to prevent wrinkling in a flat layer, and hence lower values of 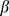 are not investigated here.

## Notes Added in Proof

Please note, the authors would like to refer the reader to the article by Eisenhoffer et al. (doi:10.1038/nature10999), which was published during the final proof stages of this publication. This paper examines cell extrusion in the crypt, and deduces that the overcrowding of cells at the crypt collar and intercrypt table leads to anoikis events, which is in agreement with the results found using the cross-sectional model.

## Supporting Information

Video S1An example of the behaviour of the epithelial monolayer when the central portion of the basement membrane is assigned a non-zero spontaneous curvature, 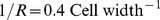 and 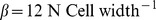. In this example, both density-dependent inhibition of mitosis and random death at the edges of the monolayer are applied, generating cell migration.(MPEG)

Video S2 A video showing how, by increasing the number of layers of stromal cells and defining a sufficiently wide monolayer, it is possible to deform an initially flat epithelial monolayer to adopt the shape of a crypt.
(MPEG)

Video S3A typical example of the cross-sectional model with parameters 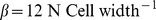, 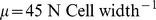, 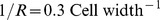. In this simulation, cell migration occurs up the vertical crypt axis where epithelial cells undergo apoptosis randomly (those cells coloured are coloured black before being removed from the simulation), or by anoikis upon losing contact with the basement membrane.(MPEG)

## References

[1] Shanmugathasan M, Jothy S (2000). Apoptosis, anoikis and their relevance to the pathobiology of colon cancer.. Path Int.

[2] Näthke I (2004). The adenomatous polyposis coli protein: the achilles heel of the gut epithelium.. Annu Rev Cell Dev Biol.

[3] Sato T, Vries RG, Snippert HJ, van de Wetering M, Barker N (2009). Single Lgr5 stem cells build crypt-villus structures in vitro without a mesenchymal niche.. Nature.

[4] Fleming ES, Zajac M, Moschenross DM, Montrose DC, Rosenberg DW (2007). Planar spindle orientation and asymmetric cytokinesis in the mouse small intestine.. J Histochem Cytochem.

[5] Quyn A, Appleton P, Carey F, Steele R, Barker N (2010). Spindle orientation bias in gut epithelial stem cell compartments is lost in precancerous tissue.. Cell Stem Cell.

[6] Nowak M, Komarova N, Sengupta A, Jallepalli P, Shih L (2002). The role of chromosomal instability in tumor initiation.. Proc Natl Acad Sci U S A.

[7] Giles R, van Es J, Clevers H (2003). Caught up in a Wnt storm: Wnt signaling in cancer.. Biochim Biophys Acta.

[8] Frisch SM, Francis H (1994). Disruption of epithelial cell-matrix interaction induces apoptosis.. J Cell Biol.

[9] Windham TC, Parikh NU, Siwak DR, Summy JM, McConkey DJ (2002). Src activation regulates anoikis in human colon tumor cell lines.. Oncogene.

[10] Van Leeuwen IMM, Byrne HM, Jensen OE, King JR (2007). Elucidating the interactions between the adhesive and transcriptional functions of *β*-catenin in normal and cancerous cells.. J Theor Biol.

[11] Reya T, Clevers H (2005). Wnt signalling in stem cells and cancer.. Nature.

[12] Korinek V, Barker N, Moerer P, van Donselaar E, Huls G (1998). Depletion of epithelial stem-cell compartments in the small intestine of mice lacking tcf-4.. Nature.

[13] van de Wetering M, Sancho E, Verweij C, de Lau W, Oving I (2002). The *β*-catenin/tcf-4 complex imposes a crypt progenitor phenotype on colorectal cancer cells.. Cell.

[14] Swat M, Kel A, Herzel H (2004). Bifurcation analysis of the regulatory modules of the mammalian G1/S transition.. Bioinformatics.

[15] Van Leeuwen IMM, Mirams GR, Walter A, Fletcher A, Murray P (2009). An integrative computational model for intestinal tissue renewal.. Cell Prolif.

[16] Meineke FA, Potten CS, Loeffler M (2001). Cell migration and organization in the intestinal crypt using a lattice-free model.. Cell Prolif.

[17] Loeffler M, Potten CS, Paulus U, Glatzer J, Chwalinkski S (1988). Intestinal cell proliferation II. Computer modelling of mitotic index data provides further evidence for lateral and vertical cell migration in the absence of mitotic activity.. Cell Tissue Kinet.

[18] Osborne JM, Walter A, Kershaw SK, Mirams GR, Fletcher AG (2010). A hybrid approach to multi-scale modelling of cancer.. Phil Trans A Math Phys Eng Sci.

[19] Fletcher AG, Breward CJW, Chapman SJ (2012). Mathematical modeling of monoclonal conversion in the colonic crypt.. J Theor Biol.

[20] Edwards CM, Chapman SJ (2007). Biomechanical modelling of colorectal crypt budding and fission.. Bull Math Biol.

[21] Drasdo D, Loeffler M (2001). Individual-based models to growth and folding in one-layered tissues: intestinal crypts and early development.. Nonlinear Anal.

[22] Nelson MR, Howard D, Jensen OE, King JR, Rose FRAJ (2010). Growth-induced buckling of an epithelial layer.. Biomech Model Mechanobiol.

[23] Hannezo E, Prost J, Joanny JF (2011). Instabilities of monolayered epithelia: shape and structure of villi and crypts.. Phys Rev Lett.

[24] Buske P, Galle J, Barker N, Aust G, Clevers H (2011). A comprehensive model of the spatiotemporal stem cell and tissue organisation in the intestinal crypt.. PLOS Comput Biol.

[25] Galle J, Loeffler M, Drasdo D (2005). Modeling the effect of deregulated proliferation and apoptosis on the growth dynamics of epithelial cell populations in vitro.. Biophys J.

[26] Schaller G, Meyer-Hermann M (2005). Multicellular tumor spheroid in an off-lattice voronoidelaunay cell model.. Phys Rev E.

[27] Drasdo D, Hoehme S, Block M (2007). On the role of physics in the growth and pattern formation of multi-cellular systems: what can we learn from individual-cell based models?. J Stat Phys.

[28] Dunn SJ, Fletcher A, Chapman S, Gavaghan D, Osborne J (2012). Modelling the role of the basement membrane beneath a growing epithelial monolayer.. J Theor Biol.

[29] Gallacher M, Mackenna BR, McKirdy HC (1973). E_ects of drugs and of electrical stimulation on the muscularis mucosae of rabbit large intestine.. Br J Pharmac.

[30] Pascal R, Kaye G, Lane N (1968). Colonic pericryptal fibroblast sheath: replication, migration and cytodifferentiation of a mesenchymal cell system in adult tissue. i. autoradiographic studies of normal rabbit colon.. Gastroenterol.

[31] Neal J, Potten C (1981). Description and basic cell kinetics of the murine pericryptal fibroblast sheath.. Gut.

[32] Pitt-Francis J, Pathmanathan P, Bernabeu MO, Bordas R, Cooper J (2009). Chaste: A test-driven approach to software development for biological modelling.. Comput Phys Commun.

[33] Heath JP (1996). Epithelial cell migration in the intestine.. Cell Biol Int.

[34] Wright N, Alison M (1984). The biology of epithelial cell populations.

[35] Alberts B, Johnson A, Lewis J, Raff M, Roberts K (2002). Molecular Biology of the Cell. 4th edition.

[36] Küppers M, Ittrich C, Faust D, Dietrich C (2010). The transcriptional programme of contactinhibition.. J Cell Biochem.

[37] Kaur P, Potten CS (1986). Cell migration velocities in the crypts of the small intestine after cytotoxic insult are not dependent on mitotic activity.. Cell Tissue Kinet.

[38] Kaur P, Potten CS (1986). Effects of puromycin, cycloheximide and noradrenaline on cell migration within the crypts and on the villi of the small intestine.. Cell Tissue Kinet.

[39] Smallwood R (2009). Computational modelling of epithelial tissues.. WIREs Syst Biol Med.

[40] Araujo RP, McElwain DLS (2004). A history of the study of solid tumour growth: the contribution of mathematical modelling.. Bull Math Biol.

[41] Gatenby RA, Maini PK (2003). Cancer summed up.. Nature.

[42] Rosenblatt J, Raff M, Cramer L (2001). An epithelial cell destined for apoptosis signals its neighbors to extrude it by an actin- and myosin-dependent mechanism.. Curr Biol.

[43] Marchiando AM, Shen L, Vallen Graham W, Edelblum KL, Duckworth CA (2011). The epithelial barrier is maintained by in vivo tight junction expansion during pathologic intestinal epithelial shedding.. Gastroenterol.

[44] Effer JC, Kee YS, Berk JM, Tran MN, Iglesias PA (2006). Mitosis-specific mechanosensing and contractile-protein redistribution control cell shape.. Curr Biol.

[45] Théry M, Bornens M (2006). Cell shape and cell division.. Curr Opin Cell Biol.

[46] Murray JD (2002). Mathematical Biology, volume 1.

[47] Shih IM, Wang TL, Traverso G, Romans K, Hamilton SR (2001). Top-down morphogenesis of colorectal tumors.. Proc Natl Acad Sci.

[48] Sangiorgi E, Capecchi M (2008). Bmi1 is expressed in vivo in intestinal stem cells.. Nat Genet.

[49] Barker N, van Es J, Kuipers J, Kujala P, van den Born M (2007). Identification of stem cells in small intestine and colon by marker gene Lgr5.. Nature.

[50] Appleton P, Quyn A, Swift S, Näthke I (2009). Preparation of wholemount mouse intestine for high-resolution three-dimensional imaging using two-photon microscopy.. J Microsc.

[51] Honda H (1983). Geometrical models for cells in tissues.. Int Rev Cystol.

[52] Ascher U, Petzold L (1998). Computer Methods for Ordinary Differential Equations and Differential-Algebraic Equations.

[53] Cheng H, Leblond CP (1974). Origin, differentiation and renewal of the four main epithelial cell types in the mouse small intestine i. columnar cell.. Am J Anat.

[54] Barker N, Ridgway RA, van Es JH, van de Wetering M, Begthel H (2009). Crypt stem cells as the cells-of-origin of intestinal cancer.. Nature.

[55] Süli E, Mayers D (2003). An Introduction to Numerical Analysis.

